# Corrosion Resistance of ALD-Deposited HfO_2_ Coatings on Stainless Steel for Biomedical Applications

**DOI:** 10.3390/ma19204363

**Published:** 2026-10-16

**Authors:** Aleksandra Seweryn, Ewa Ura-Binczyk, Bartłomiej S. Witkowski, Wojciech Woźniak, Dariusz M. Jarzabek, Krzysztof Kulikowski, Hanna Konopacka, Anna Wolska, Marek Godlewski, Marcin T. Klepka

**Affiliations:** 1 Institute of Physics, Polish Academy of Sciences, Aleja Lotników 32/46, 02-668 Warsaw, Poland; bwitkow@ifpan.edu.pl (B.S.W.); wwozniak@ifpan.edu.pl (W.W.); wolska@ifpan.edu.pl (A.W.); godlew@ifpan.edu.pl (M.G.); mklepka@ifpan.edu.pl (M.T.K.); 2 Faculty of Materials Science and Engineering, Warsaw University of Technology, Woloska 141, 02-507 Warsaw, Poland; ewa.ura@pw.edu.pl (E.U.-B.); krzysztof.kulikowski@pw.edu.pl (K.K.); 3 Institute of Fundamental Technological Research, Polish Academy of Sciences, Pawinskiego 5B, 02-106 Warsaw, Poland; djarz@ippt.pan.pl (D.M.J.); hkonop@ippt.edu.pl (H.K.); 4 Faculty of Mechatronics, Institute of Micromechanics and Photonics, Warsaw University of Technology, Sw. A. Boboli 8, 02-525 Warsaw, Poland

**Keywords:** XPS, ALD, stainless steel, bone implant coating, electrochemical measurements

## Abstract

This study investigated the electrochemical protection provided by amorphous hafnium thin films deposited on austenitic stainless steel by atomic layer deposition technology that guarantees control over the physicochemical properties of deposited coatings and processes repeatability. Furthermore, ALD enables conformal coating of three-dimensional shapes. The corrosion resistance of these coatings was evaluated under conditions simulating physiological environments, using potentiodynamic polarization and electrochemical impedance spectroscopy. Furthermore, X-ray photoelectron spectroscopy, scanning electron microscopy, and atomic force microscopy were employed to analyze the chemical stability, surface condition, and morphology of the films prior to and following electrochemical testing. Potentiodynamic polarization tests demonstrated that uncoated SS316L provided insufficient corrosion resistivity, whereas samples coated with ALD-grown hafnium dioxide thin films possessed barrier properties, exhibited a pronounced passive region and a reduction in anodic and cathodic current densities by up to three orders of magnitude. Electrochemical impedance spectroscopy results further verified the development of a compact, highly resistive dielectric interface. X-ray photoelectron spectroscopy analysis demonstrated that the films exhibited chemical stability following corrosion. SEM images showed that after electrochemical tests on stainless steel, significant surface degradation was observed, whereas the hafnium dioxide-coated surfaces remained unchanged. The findings suggested that films with a thickness of ≈100 nm act as effective barriers to corrosive ions. These results highlight the potential of ALD-deposited amorphous hafnium dioxide thin films as protective coatings against corrosion and present promising prospects for future biomedical applications.

## 1. Introduction

Metal implants are frequently used in the treatment of bone fractures owing to their excellent mechanical properties. Among the most common materials used for orthopedic devices, such as screws, fixation plates, and bone replacement scaffolds, are titanium, cobalt–chromium alloys, and stainless steel 316 L (SS316L). Despite their advantages, these metallic implants are prone to corrosion when exposed to physiological environments, which can potentially affect their structural integrity and compatibility with the biological tissues.

The physiological environment is characterized by an ionic medium with a pH close to 7.4 and a temperature ≈ 37 °C [1], which facilitates surface degradation. Consequently, corrosion releases metal ions that can accumulate in tissues, leading to local inflammation, implant loosening, and systemic adverse effects [2,3,4,5]. Although a thin native oxide layer naturally forms on metallic implant surfaces and initially acts as a protective barrier, its limited thickness and porosity compromise long-term stability, allowing for ongoing ion release. Continuous ion release can trigger local irritation, metallosis, and chronic inflammatory responses [6].

Although titanium alloys are generally considered biocompatible, reports of their potential toxicity warrant caution. These concerns primarily stem from the release of alloying elements into the body and the possible toxicity of titanium dioxide [7,8,9], underscoring the need for continued investigation into alternative materials.

SS316L is a cost-effective alternative to titanium for long-bone implants, such as hip prostheses, owing to its superior strength and ductility. However, SS316L is susceptible to fatigue cracking, often beginning at locations of pitting corrosion [10]. In physiological settings, pitting results from localized biochemical interactions.

To protect metallic surfaces from harsh ionic species present in physiological fluids, implant coatings have been proposed. Among the established commercial methods are the anodization of titanium for titanium implants [11] and the use of magnetron sputtering to apply protective and bioactive coatings [12]. Despite the widespread use of magnetron sputtering, its directional nature poses a challenge in achieving uniform coatings on intricate three-dimensional shapes, a drawback that is becoming more significant with the rise in modern patient-specific implant designs.

Atomic layer deposition (ALD) is a well-established technique for producing conformal thin films [13] and is worth considering as an alternative to chemical vapor deposition or physical vapor deposition. Physical vapor deposition (PVD) involves the physical ejection and condensation of a source material-through sputtering or evaporation-onto a substrate, which typically requires line-of-sight deposition and offers limited conformation for complex geometries. Chemical vapor deposition (CVD) involves the reaction or decomposition of gaseous precursors on the substrate surface, forming a solid layer, enabling good step coverage and high deposition rates. It requires high deposition temperatures. In CVD, the precursors react in the vapor phase, resulting in less control over the chemical composition and consistent film quality. ALD is an optimal technique for deposition of ultrathin films due to its simplicity, repeatability, and excellent control over the composition, thickness, and conformation of the layer at the atomic level. The wide deposition temperature range allows for processes from room temperature to 500 °C, depending on the chosen reactor [14,15].

Originally developed for the electronics industry [15], ALD enables high-quality film growth and has broad application potential. The chemical composition of ALD films is highly versatile [16,17] and can be controlled with atomic-scale precision. Film growth proceeds via self-limiting surface reactions, in which precursor ligands are chemisorbed to reactive surface sites. The resulting films are conformal, strongly adherent, and form continuous coatings that faithfully replicate underlying geometries, even on complex three-dimensional structures [13].

Numerous research teams have demonstrated the corrosion protection capabilities of transition-metal oxide films deposited by ALD and conducted an extensive investigation into the corrosion resistance of ALD thin films when applied to metallic substrates [18,19,20,21]. Magnesium alloys with a 100 nm ZrO_2_ coating exhibited better corrosion resistance than those with the same thickness of TiO_2_ [18,20]. Additionally, HfO_2_ coatings on magnesium alloys offer slightly better barrier performance than ZrO_2_ [19]. In a direct comparison of deposition methods, Peron et al. found that TiO_2_ coatings on porous substrates exhibited superior surface integrity and adhesion when deposited by ALD, in contrast to magnetron sputtering, which resulted in enhanced corrosion resistance [21].

Oxide coatings grown by ALD at low temperatures were assessed for their anticorrosion properties on copper substrates. Among the frequently examined oxides, including Al_2_O_3_, TiO_2_, ZnO, ZrO_2_, and HfO_2_, the latter showed the highest level of protection [22]. Thin coatings of Al_2_O_3_ and HfO_2_, with thicknesses ranging from 25 to 50 nm, demonstrated excellent barrier properties. However, while Al_2_O_3_ coatings deteriorated after 21 days in a NaCl solution, HfO_2_ coatings maintained their structural integrity and barrier effectiveness during the same period [22]. ALD-deposited HfO_2_ films are well known for their chemical stability and resistance to corrosion. For instance, Dinu et al. observed that HfO_2_ layers deposited by ALD enhanced the barrier performance of Ti(N,O) coatings on 304L stainless steel created through cathodic arc evaporation [23]. Likewise, Li et al. discovered that transition-metal oxides obtained by ALD on 304 stainless steel displayed high chemical stability and excellent corrosion resistance under both acidic and alkaline conditions [17].

Although these findings are promising, the corrosion resistance provided by ALD-deposited HfO_2_ films on SS316L remains insufficiently understood. This knowledge gap is significant because the performance of nanocoatings is greatly affected by the properties of the substrate, including surface chemistry, roughness, and microstructure, as well as deposition parameters such as growth temperature, precursor chemistry, and film thickness.

To evaluate the electrochemical properties of SS316L coated with hafnium films compared to pure SS316L, potentiodynamic polarization (PP) and electrochemical impedance spectroscopy (EIS) were applied.

Because corrosion processes originate at the material surface, advanced surface-sensitive techniques are essential for their detection. In this context, scanning electron microscopy (SEM), X-ray photoelectron spectroscopy (XPS), and atomic force microscopy (AFM) are powerful complementary methods that provide detailed information on the surface composition and chemical states and thus complement electrochemical and structural analyses. XPS data can therefore aid the interpretation of corrosion phenomena and the evaluation of coating–substrate interfaces [24,25].

This study is part of a larger investigation into the extensive applications of ALD-deposited HfO_2_ coatings for biomedical implants. Earlier studies [26] highlighted the biological significance of low temperature deposited amorphous HfO_2_ nanolayers [27] in promoting bone regeneration. This study advances this research by assessing the electrochemical stability of amorphous HfO_2_ on SS316L in corrosive environments that mimic physiological conditions to evaluate their suitability as multifunctional coatings for bone implants.

## 2. Materials and Methods

### 2.1. ALD Thin Film Preparation

HfO_2_ thin films were applied to silicon substrates for thickness evaluation and on SS316L disks for structural and functional analysis. These disks were produced from commercially sourced SS316L rods manufactured by Viraj Profiles Private Limited, Tarapur, India, and supplied in accordance with ASTM A276/A276M-24a, ASTM International (in addition to iron: C 0.026, Mn 1.53, Si 0.55, S 0.007, P 0.037, Cr 16.74, Ni 10.01, Cu 0.34, Mo 2.01, Ti 0.002, Co 0.19, N 0.071 (wt.%)). Cylindrical samples, measuring ⌀ 15 × 2 mm, were crafted through machining and then underwent mechanical surface treatment to achieve the required surface quality. The samples were ground using water-lubricated abrasive papers with grit sizes from #120 to #2400, and subsequently polished with a 1 μm diamond suspension to achieve a mirror-like finish with a surface roughness of less than Ra = 0.02 μm. All substrates were cleaned in an ultrasonic bath (Sonic-3, Polsonic, Warsaw, Poland) at 40 °C, undergoing sequential rinsing for 5 min each in acetone, 2-propanol, and deionized water (repeated twice), and then dried with a stream of high-purity nitrogen (5N). Film deposition was performed using ALD in a Savannah-100 reactor (Cambridge NanoTech Inc., Cambridge MA, USA). Tetrakis(diethylamido) hafnium (TDMAH; Hf[N(CH_3_)_2_]_4_, CAS No. 19782-68-4; Sigma Aldrich, Saint Louis, MO, USA) was used as the hafnium precursor, while deionized water acted as the oxygen source. High-purity nitrogen (6N) (from Messer Group, Warsaw, Poland) served as the carrier and purging gas. Each ALD cycle included a TDMAH pulse, a purge step, a water vapor pulse, and another purge step. The pulse and purge sequence was set at 4 ms–8 s–2 ms–22 s. The 660 ALD cycles were adjusted to produce thin films deposited at a temperature of 90 °C.

### 2.2. Test Equipment

Reflectometry was used to determine the thickness of the deposited layers. Measurements were performed using a NanoCalc 2000-UV/VIS system (Micropack GmbH, Ostfildern, Germany) equipped with a dedicated analysis software Nanocalc232, Ostfildern, Germany.

XPS was conducted using a Scienta MX 650 X-ray source which emitted monochromatic Al K_α_ radiation (h*ν* = 1486.6 eV) at 150 W eqquiped with a Scienta R4000 hemispherical analyzer, Scienta Omicron, Uppsala, Sweden. The energy resolution, assessed using the full width at half maximum (FWHM) of the Au 4f_2/7_ reference line under the same conditions, was 0.65 eV. The samples were examined without charge neutralization. The energy scale was calibrated by setting the C 1s peak at 285 eV. CasaXPS software, version 2.3.26PR1.0 was used to deconvolve all spectra. Peak fitting utilized a combination of Gaussian and Lorentzian line shapes with the composition of 60% Gaussian and 40% Lorentzian, and the background was modeled using the Shirley function.

SEM was conducted using a Hitachi SU70 microscope, with spatial resolution of 1 nm and equipped with an electron detector. Images were captured at an accelerating voltage of 15 kV.

AFM measurements were performed to assess the surface uniformity and nanoscale roughness variations due to corrosion exposure. The surface topography was evaluated using a Nanosurf Flex AFM, Nanosurf GmbH, Langen, Germany operating in the contact mode with constant-force feedback. The measurements utilized an HQ:CSC17/Al BS silicon cantilever probe from MikroMasch, MikroMasch Europe, Wetzlar, Germany, featuring an Al reflective back-side coating. This probe had a nominal spring constant of 0.18 N/m, resonance frequency of 13 kHz, and tip radius of less than 8 nm. During the measurements, the setpoint force was maintained at 400 mV. Surface scans covered areas of 50 × 50 μm^2^ with a resolution of 500 × 100 points and were conducted under standard laboratory conditions. For each sample, five distinct surface regions were analyzed, and five line profiles were extracted from each region, resulting in a total of 25 profiles per sample. The arithmetic mean roughness (Ra), corresponding to the arithmetic average of the absolute deviations of a surface profile from its mean (centerline) plane, was derived from these profiles and presented as the mean value ± standard deviation. Opensource Gwyddion software, ver. 2.55 was used for AFM image processing and the roughness analysis.

Electrochemical measurements were conducted using PP tests with an Autolab PGSTAT302N (Metrohm, Herisau, Switzerland) potentiostat/galvanostat. The tests were performed in a 0.9% NaCl solution, which was naturally aerated and unstirred at a constant temperature of 37 °C. A standard three-electrode configuration was employed, consisting of a platinum sheet as the counter electrode (CE), a silver chloride electrode (Ag|AgCl|Cl^−^) as the reference electrode (RE), and the sample serving as the working electrode (WE). Before conducting the electrochemical measurements, the specimens were placed in the electrolyte for 1 h under open-circuit conditions. Potentiodynamic polarization (PP) measurements were performed to evaluate the electrochemical corrosion behavior of uncoated and HfO_2_-coated SS316L, including the corrosion potential, current density, passive behavior, and pitting response. The PP scans commenced 0.15 V below the E_*OCP*_, with a scan rate of 1 mV/s, and concluded when the current density reached 1 mA·cm^−2^.

In EIS experiments, a sinusoidal wave disturbance of ±10 mV relative to E_*OCP*_ was used. The frequency range extended from 100 kHz to 10 mHz, capturing 10 data points per decade. NOVA 2.0 software was employed for both measurements and equivalent circuit fitting, maintaining the fitting error below 5%. Each measurement was conducted at least three times.

## 3. Results

**Figure 1:**
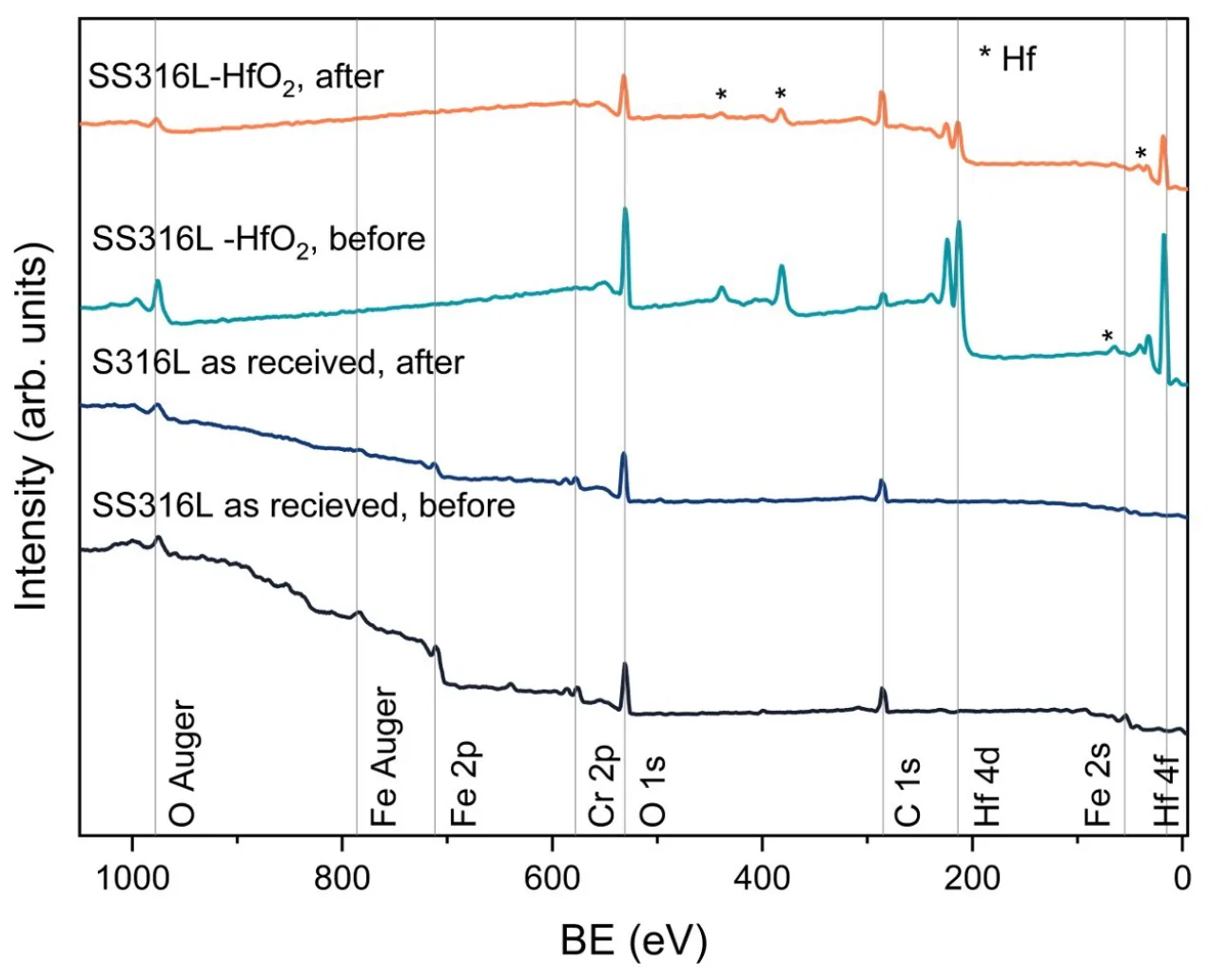
XPS survey spectra of SS316L and HfO_2_ films deposited on SS316L surface before and after electrochemical measurements. The asterisk indicates a signal originating from hafnium.

Reflectometry measuerments indicated that thin films with a thickness of ≈100 nm were obtained using the ALD technique. XPS analysis provided insights into the quality of HfO_2_ coatings applied to SS316L substrates. These findings were compared to HfO_2_ coatings on Si substrates, which served as a reference. The examined surfaces coated with HfO_2_ revealed the presence of only Hf, O, and C [28] (Figure 1). The estimated Hf:O ratio was 0.54, suggesting that the HfO_2_ was nearly stoichiometric.

**Figure 2:**
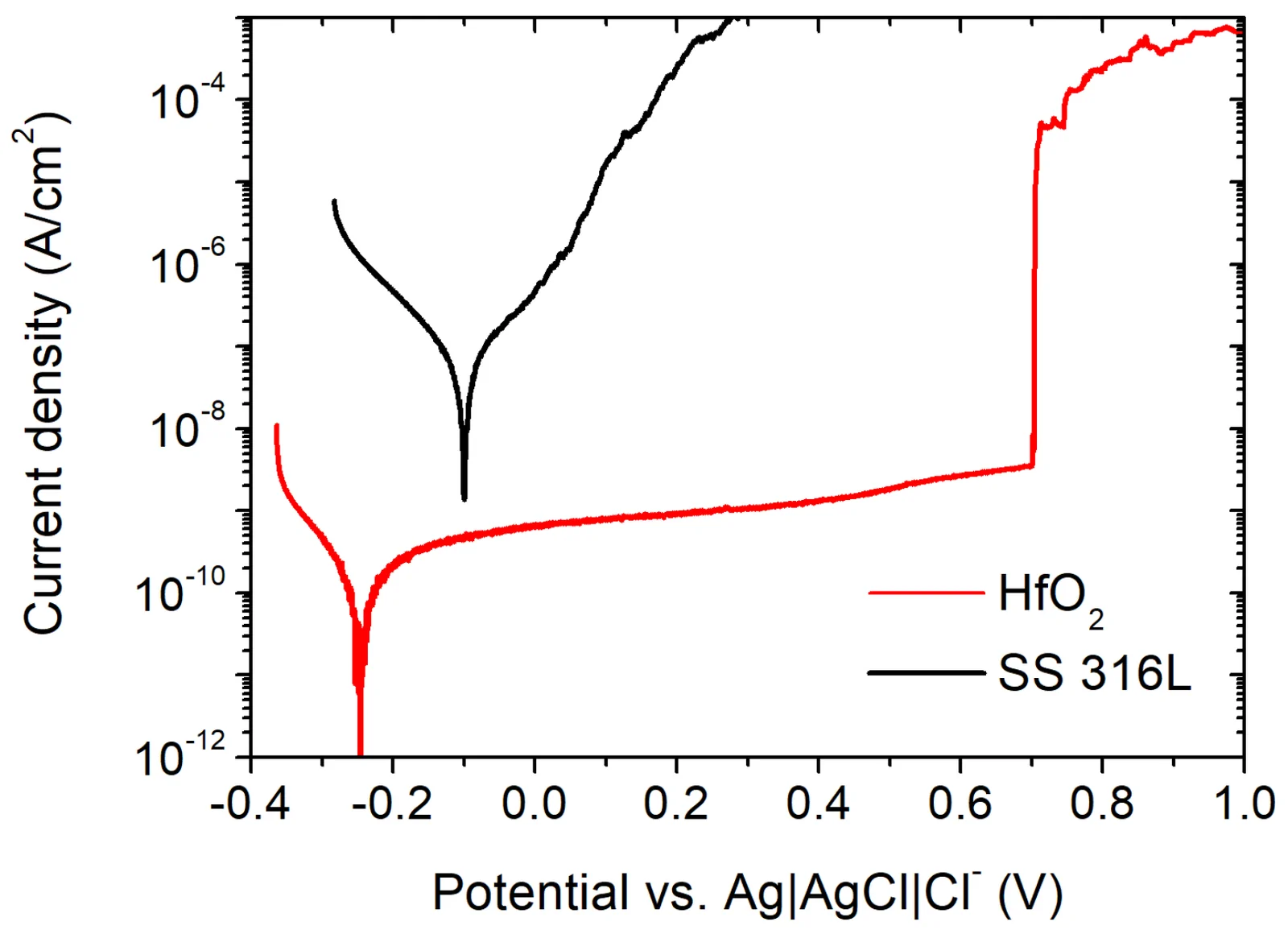
PP curves obtained for SS316L and for HfO_2_ coatings in naturally aerated 0.9% NaCl at the temperature of 37 °C.

PP measurements were conducted to assess the corrosion characteristics of HfO_2_ coatings on SS316L in a 0.9% NaCl solution at 37 °C. Figure 2 shows the typical polarization curves, while Table 1 presents the average electrochemical parameters derived from all recorded curves. The uncoated reference sample showed active corrosion, with a swift rise in anodic current on the SS316L surface and no passive region, highlighting the inadequate barrier function of the native oxide formed on pure SS316L. Conversely, the HfO_2_-coated sample exhibited a distinct passive plateau and a notable decrease in both anodic and cathodic current densities, reduced by up to three orders of magnitude. The abrupt increase in anodic current density observed for the HfO_2_-coated SS316L sample at potentials close to 0.7 V was related to the local breakdown of the protective state and the subsequent initiation and propagation of pitting corrosion. The stable current density within the passive range confirmed the pitting resistance provided by the HfO_2_ coating.

**Figure 3:**
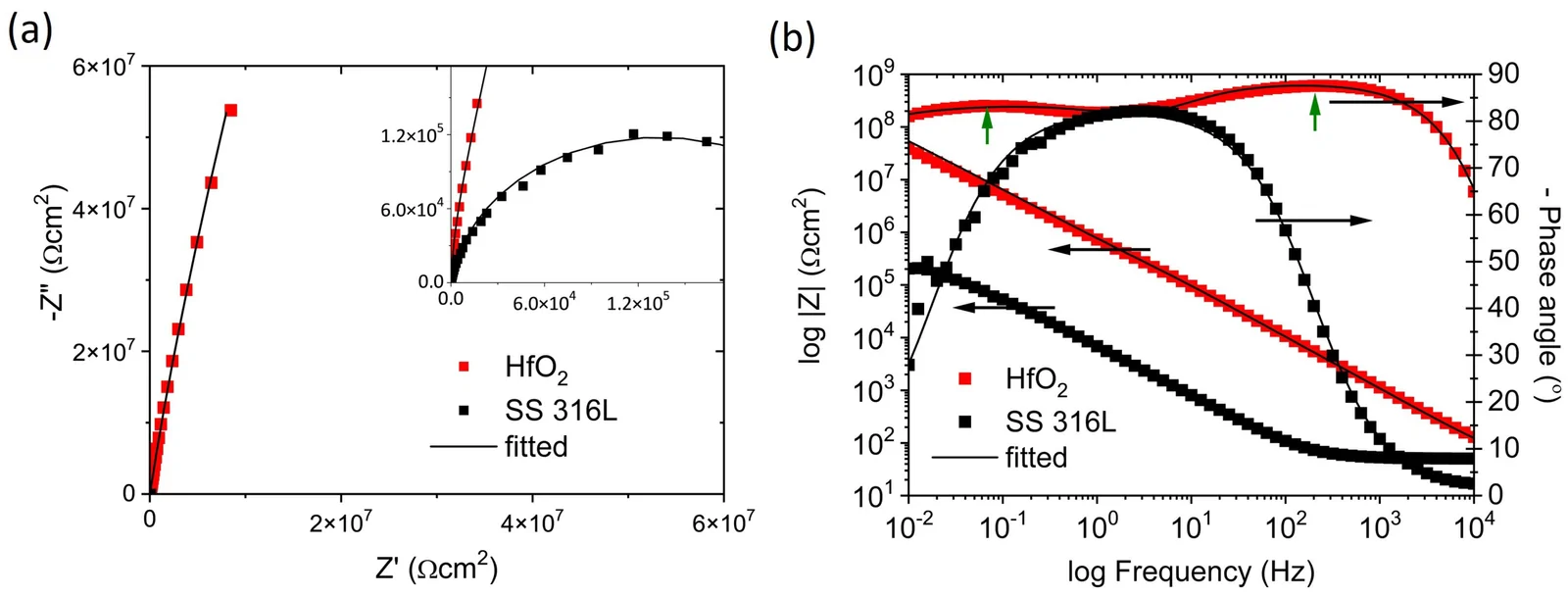
Nyquist (**a**) and Bode (**b**) plots obtained for SS316L and for HfO_2_ coating sample in naturally aerated 0.9% NaCl at the temperature of 37 °C.

EIS was conducted to deepen the understanding of corrosion processes. Figure 3 shows the Nyquist and Bode plots obtained after immersing the samples for 1 h in 0.9% NaCl. The Nyquist plots revealed capacitive characteristics of the samples. Remarkably, the impedance loops for the sample with a HfO_2_ coating exhibited values that were several orders of magnitude higher than those of the uncoated sample, indicating enhanced corrosion resistance (Figure 3a). In the Bode plots (Figure 3b), the uncoated sample showed a single time constant and a relatively narrow high-phase-angle plateau, indicating a predominantly capacitive response associated with the passive surface. In contrast, the HfO_2_-coated sample exhibited a broader phase-angle plateau at values approaching 90°, indicating a predominantly capacitive and highly insulating response over a broad frequency range. In addition, two distinct maxima can be observed in the phase-angle versus log-frequency plot (marked with two green arrows), corresponding to two characteristic time constants of the coated system. These two time constants are consistent with the two-time-constant equivalent circuit used for the HfO_2_-coated sample and are associated with the electrochemical response of the HfO_2_ coating and the underlying coating/substrate interface, respectively.

**Figure 4:**
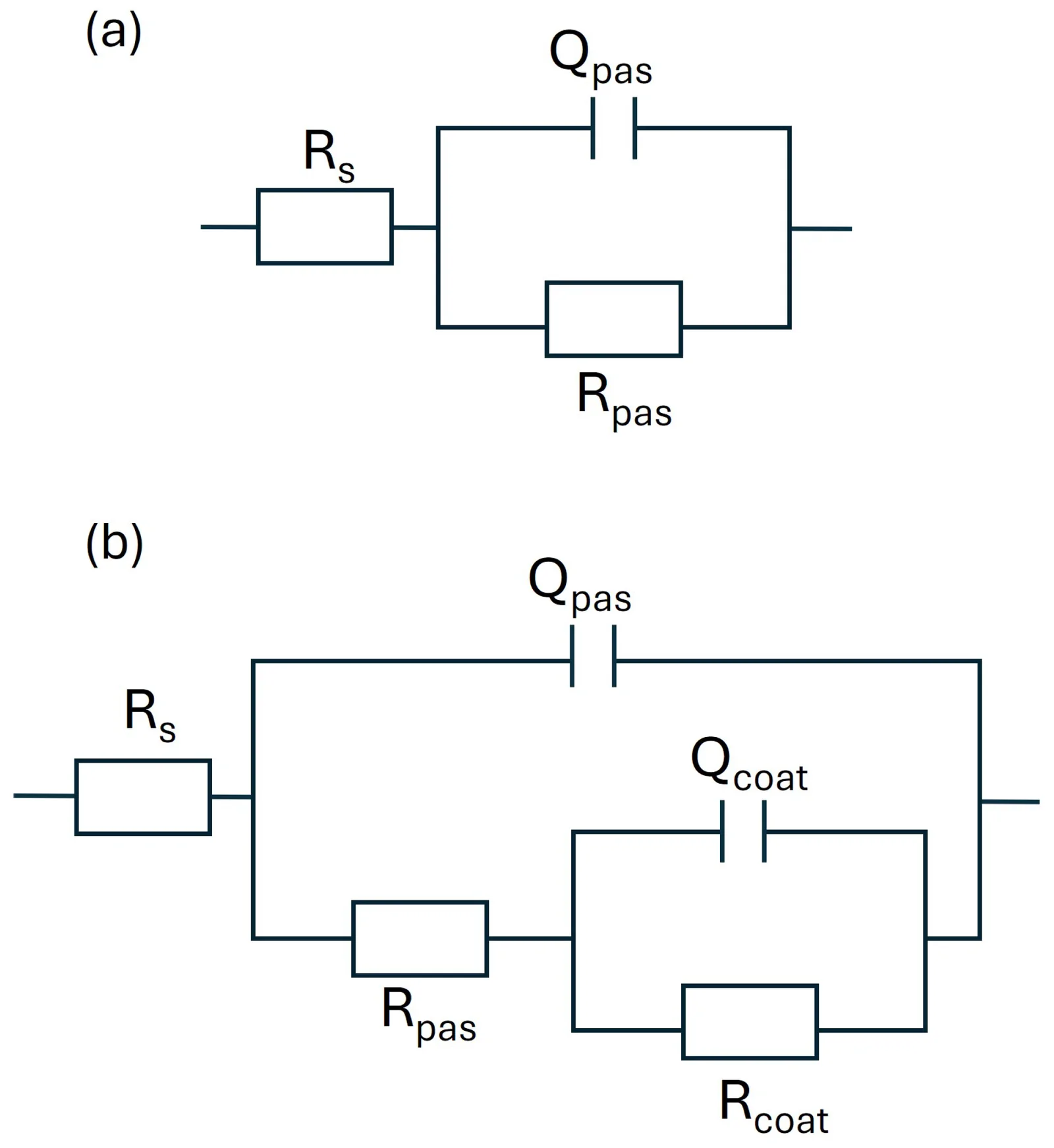
Equivalent circuit: one-time constant (**a**) and two-time constant (**b**) model.

The impedance measurements were analyzed using electrochemical equivalent circuits (EECs), as depicted in Figure 4. For uncoated samples, a Randle circuit with a single time constant was employed (Figure 4a), whereas coated samples utilized a circuit with two time constants, incorporating constant phase elements (CPEs) (Figure 4b) [29].

The parameters obtained from the EIS modeling are listed in Table 2. The solution resistance ($R_{S}$) was consistently around 50 Ω·cm^2^ for all samples, indicating comparable electrolyte resistance and supporting the comparison of interfacial characteristics. The resistance of the passive layer ($R_{\mathrm{pas}}$) showed a significant increase in the HfO_2_-coated sample, reaching 0.63 MΩ·cm^2^, compared to 0.23±0.01 MΩ·cm^2^ for the uncoated SS316L. This increase indicates improved barrier properties of the HfO_2_ film and its contribution to the enhanced corrosion resistance. The constant phase element (CPE) parameter for the passive layer, $Y_{0-\mathrm{pas}}$, further supports this observation. The coated sample demonstrated a significantly lower CPE admittance parameter $Y_{0}$ of $0.19\pm 0.02\;\mu S\;\cdot \;s^{n}\;\cdot \;{cm}^{-2}$, compared to $28.3\pm 0.5\;\mu S\;\cdot \;s^{n}\;\cdot \;{cm}^{-2}$ for the uncoated sample. The lower $Y_{0}$ value, together with the substantially higher resistance, is consistent with the formation of a more resistive and less electrochemically active interfacial layer after HfO_2_ deposition. The lower $Y_{0}$ value and the high $n_{\mathrm{pas}}$ value further indicate a more ideal capacitive response of the coated interface. The exponent $n_{\mathrm{pas}}$ approached unity in the HfO_2_-coated sample (0.97±0.01), indicating similar behavior to that of an ideal capacitor. This response is consistent with a relatively homogeneous and continuous dielectric barrier formed by the HfO_2_ coating.

These findings are consistent with the results reported by Li et al. for ALD-HfO_2_ deposited on 3ss316L. The authors observed a clear increase in the overall impedance of HfO_2_-coated stainless steel compared to the uncoated substrate, accompanied by a substantial increase in polarization resistance and a marked decrease in corrosion current density [17]. Although the CPE parameters were not directly compared in that study, the increased impedance observed after HfO_2_ deposition is consistent with the more resistive and dielectric interfacial response observed in the present work.

**Figure 5:**
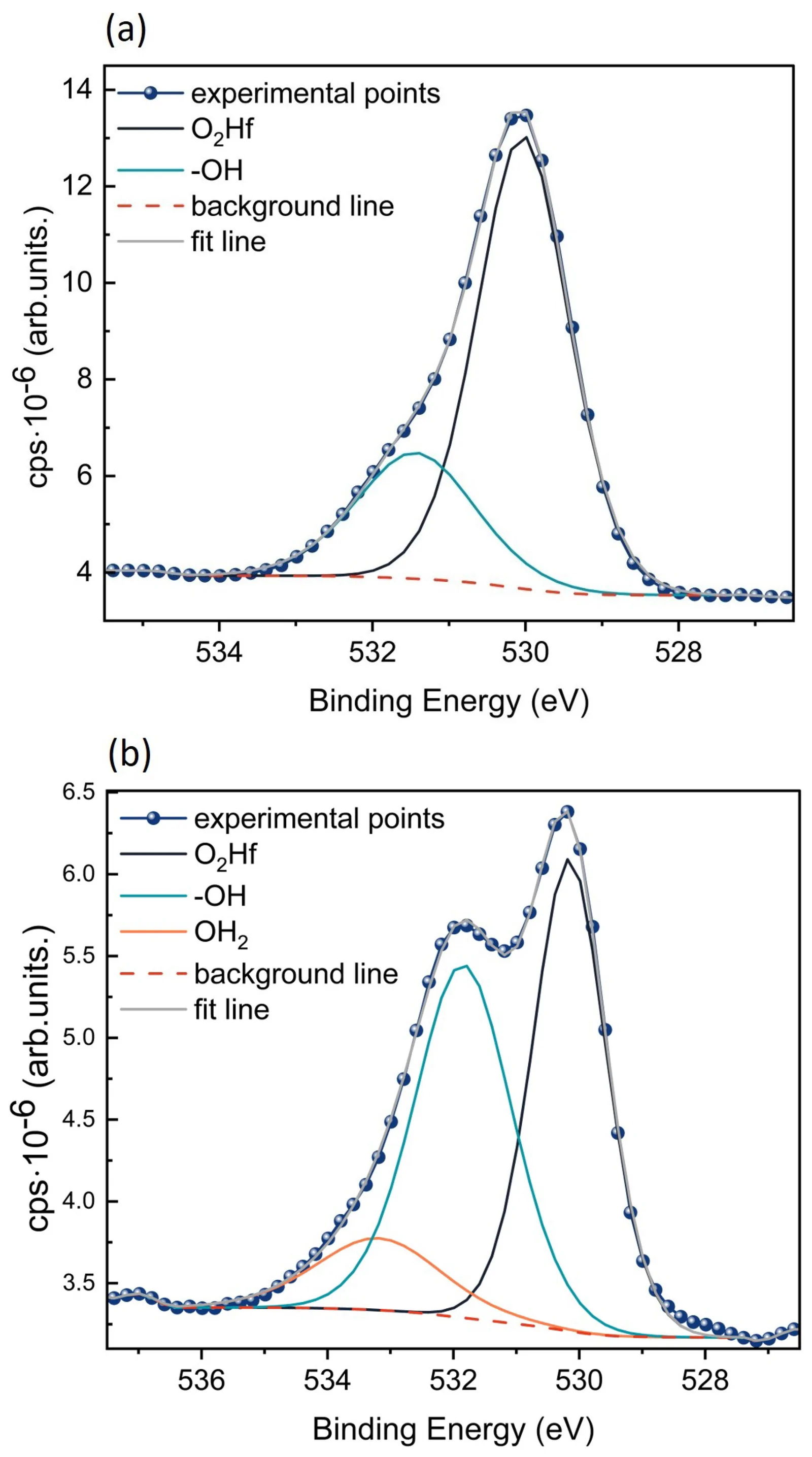
The XPS results of O 1s line taken from HfO_2_ coating before (**a**) and after electrochemical measurements (**b**).

XPS measurements were conducted before and after the electrochemical testing. Figure 5 presents the high-resolution XPS spectra of the O 1s line captured before (Figure 5a) and after (Figure 5b) the electrochemical evaluations. The O 1s lines of samples before are shown in Figure 5a and were deconvoluted into components: The primary O 1s peak, located at a binding energy ≈530.0 eV [28], corresponds to lattice oxygen atoms. The secondary component of the O 1s line, detected at a binding energy ≈532 eV, is linked to hydroxyl (–OH) groups (Table 3), which typically form during ALD [16]. The third component, which appeared only in the sample after electrochemical testing (Figure 5b), was found around a BE of ≈533 eV and was attributed to adsorbed H_2_O and/or organic species on the surface, in line with the table value [28].

The Hf 4f line was deconvoluted by considering spin–orbit splitting, with a binding energy (BE) ≈ 16.6 eV, a full width at half maximum (FWHM) of about 1.1 eV, and a spin–orbit separation Δ = 1.7 eV. These parameters align with the table values reported for HfO_2_ [28]. The Hf 4f line spectra remained similar before and after the electrochemical tests.

**Figure 6:**
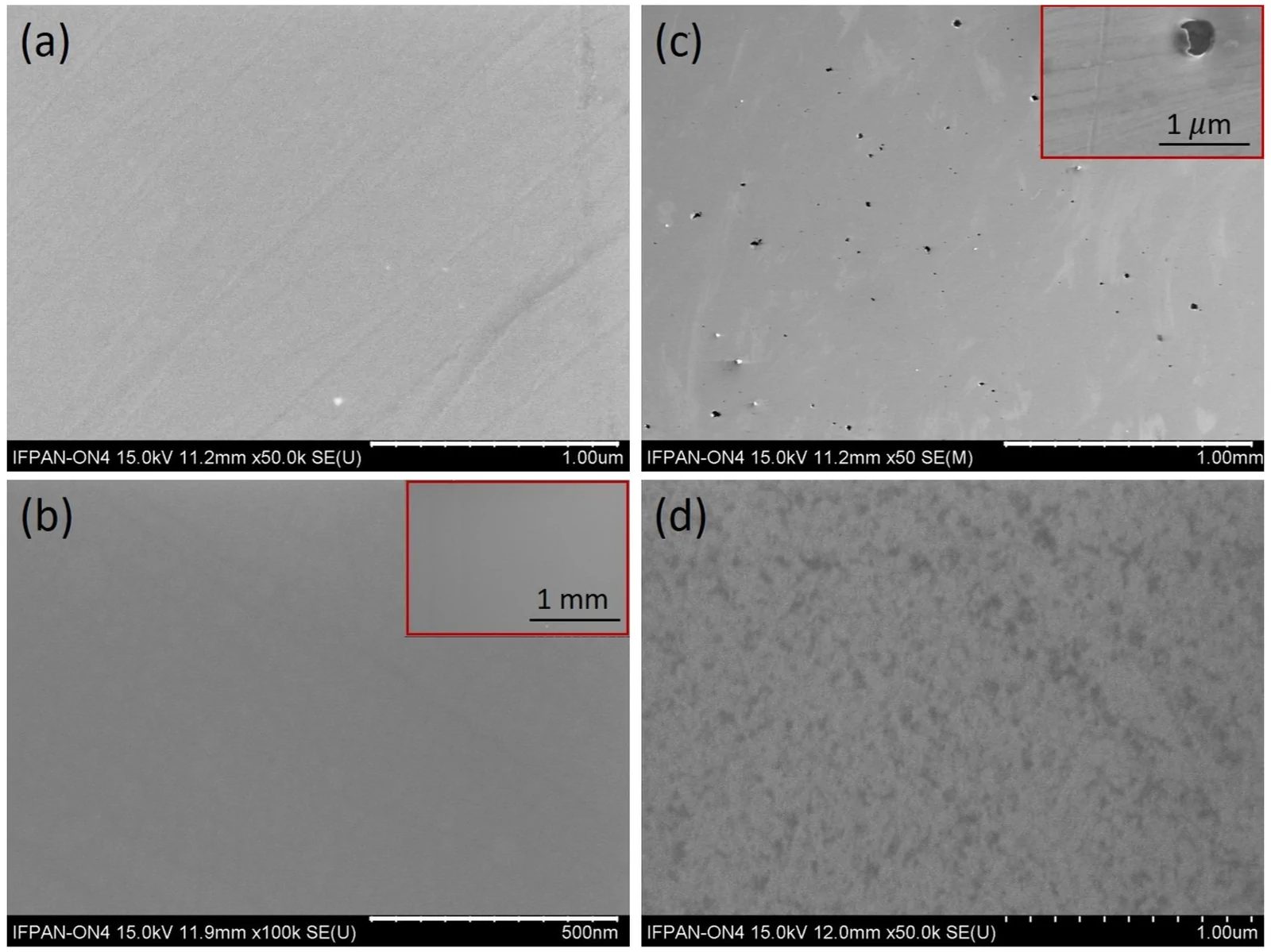
SEM images showing the surface before electrochemical measurements of SS316L (**a**) and HfO_2_; the image at lower magnification (inset) shows the continuity of the surface (**b**); electrochemical measurements of SS316L, with corrosion-induced surface damage at higher magnification (inset) (**c**) and HfO_2_ coating (**d**).

SEM images of the pure SS316L sample captured before (Figure 6a) and after (Figure 6b) electrochemical testing revealed the presence of cracks caused by corrosion. The SS316L disks were entirely covered with a HfO_2_ film. Initially, the surface of the HfO_2_-coated sample was smooth and free of visible cracks before undergoing electrochemical testing (Figure 6b). After testing, minor inhomogeneities appeared; however, no apparent damage to the coating was observed (Figure 6d).

**Figure 7:**
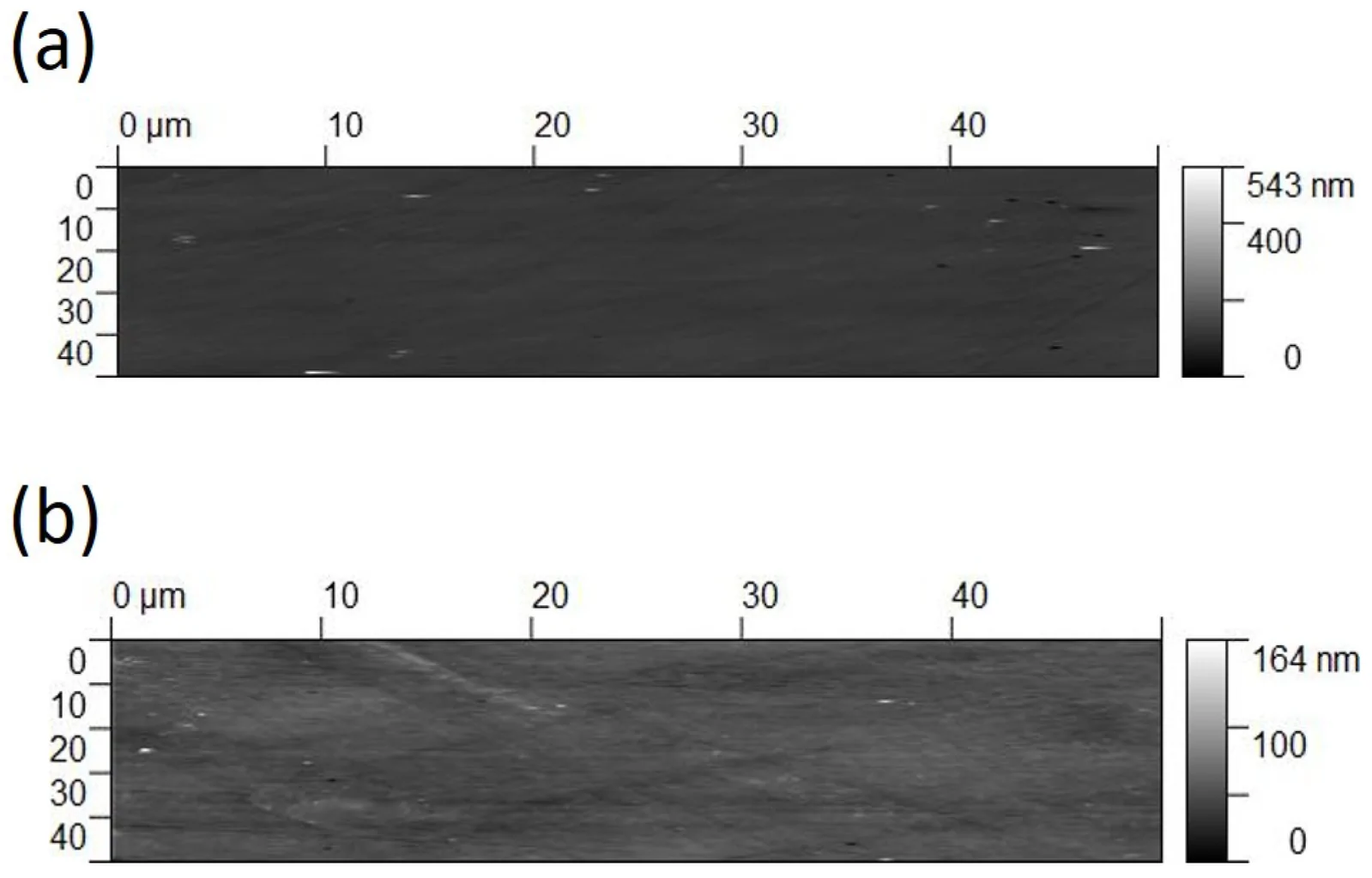
AFM images showing the HfO_2_-coated surface: before (**a**) and after electrochemical measurements (**b**).

The surface roughness of the HfO_2_-coated sample was measured at Ra 1.97 ± 0.28 nm, reflecting a smooth and featureless surface morphology, aligning with the SEM findings. However, after electrochemical testing, the roughness of the HfO_2_-coated sample increased to Ra 2.75 ± 0.42 nm. Figure 7 presents representative AFM maps.

## 4. Discussion

Continuous HfO_2_ films were deposited on SS316L disks. This type of conformal substrate coating is a recognized feature of films produced by ALD [13]. Analysis through both SEM and XPS demonstrated that the chosen ALD precursors, TDMAH and HfO_2_, interacted efficiently with the SS316L surface, facilitating the formation of films ≈100 nm thick. Moreover, the XPS survey spectra matched those of HfO_2_ films on silicon, with a quality comparable to that grown on silicon substrates used as a reference. Additionally, no XPS signals from SS316L were observed in the HfO_2_-coated samples, either prior to or following electrochemical testing (Figure 1), which further verified the chemical stability of the coatings. This suggests that the HfO_2_ coatings on SS316L possess high integrity and continuity at the atomic level, a finding also supported by SEM analysis (Figure 6b,d).

The HfO_2_-coated SS316L amorphous [1] was used to assess their effectiveness as barriers against corrosion. A decreased current density is generally associated with a reduced corrosion rate and enhanced corrosion resistance [30]. The corrosion potential, in contrast, reflects the thermodynamic state of the electrode/electrolyte interface and does not necessarily shift toward more positive values when the corrosion resistance is improved. In the present study, the HfO_2_-coated sample demonstrated enhanced corrosion resistance owing to the ALD coating (Figure 2), despite the shift in the corrosion potential toward more negative values. A similar behavior has been reported for ALD-HfO_2_-coated 304 stainless steel, where the corrosion potential shifted toward more negative values while the current density decreased substantially [17]. Previous studies have also reported that magnesium alloys coated with ALD TiO_2_ and ZrO_2_ experienced a reduction in current density by more than tenfold [19,21], whereas the HfO_2_ coating on SS316L in this study achieved a reduction of up to a thousandfold. This significant decrease in current density is expected to reduce the release of metal ions. These findings indicate the potential use of HfO_2_ coating as a barrier layer for bone implants to mitigate the risks of metallosis and chronic inflammation associated with uncoated SS316L implants [1,2,3,4,5,6].

The constant phase element (CPE) provides a more precise representation of the non-ideal capacitive behavior caused by surface irregularities, such as porosity [31] and roughness [32]. The HfO_2_ layer’s low CPE admittance parameter ($Y_{0\text{-}\mathrm{coat}}$) of 0.15 $\mu S\;\cdot \;s^{n}\;\cdot \;{\mathrm{cm}}^{-2}$ is particularly revealing: such low $Y_{0}$ values suggest diminished interfacial activity and a more dielectric (less conductive) coating, consistent with an effective barrier layer. Collectively, these findings suggest that the amorphous film deposited at low temperatures provides a continuous and effective dielectric barrier. Clinically, this may contribute to a more durable barrier against ion release, potentially reducing the risk of implant-related inflammation or metallosis.

The integrity of the corrosion barrier was evaluated using XPS because of its non-destructive properties and high surface sensitivity [24,25]. It was anticipated that the XPS surface characteristics would remain unchanged before and after the electrochemical tests. Additionally, the HfO_2_ film was nearly ideal in terms of stoichiometry and demonstrated the stability of the Hf 4f line. This stoichiometric evaluation was further corroborated by the analysis of the O 1s spectrum.

An increase in the atomic concentration following electrochemical testing suggests a heightened surface reactivity with the aqueous environment. Generally, the presence of –OH groups after electrochemical measurements may imply an increased surface reactivity and a potential decrease in electrochemical resistance. Nonetheless, the survey spectra obtained for the HfO_2_-coated sample, both before and after the experiment, indicated no diffusion of metallic ions from SS316L to the surface. The stability of the Hf 4f peak, as evidenced by the high-resolution XPS spectra, along with the absence of substrate-related species, implies that there was no significant surface degradation. Consequently, the increase in the O 1s hydroxyl component is linked to the interactions between HfO_2_ and the electrolyte solution on the surface. The third component of the O 1s peak, detected at a binding energy ≈533 eV (Figure 5b), is associated with adsorbed H_2_O and/or organic species on the surface [28] following the electrochemical measurements. Considering that the electrochemical experiments were performed in an aqueous environment, the presence of residual water or organic species after testing was anticipated.

The lattice-related component of the O 1s line showed no change following the electrochemical measurements, maintaining a BE of ≈530 eV and an FWHM ≈1.4 eV, consistent with the values recorded for the sample before these measurements. Unlike the SS316L reference, SEM observations verified that the coated surfaces remained stable. Figure 6c illustrates the corrosion of the SS316L surface, whereas Figure 6d shows that the coated sample remained intact after the electrochemical testing. Additionally, the minimal change in surface roughness before and after the measurements reinforces the main conclusion of this study: the corrosion resistance of ALD-deposited HfO_2_ films is largely determined by the microstructural integrity and absence of pinholes in the amorphous HfO_2_ film [17] applied to the SS316L surface. In addition to the previously documented osteoregenerative properties of ALD-deposited HfO_2_ [26,33], the current findings on corrosion barrier performance suggest that amorphous HfO_2_ serves as a versatile surface modification, enhancing the early osteogenic response while safeguarding the SS316L substrate from degradation in physiological environments.

## 5. Conclusions

Coating SS316 with amorphous HfO_2_ thin films deposited by ALD significantly enhanced the corrosion resistance of austenitic stainless steel in environments analogous to physiological conditions. Electrochemical tests, surface analyses, and XPS studies consistently demonstrated that the HfO_2_ layer exhibited barrier properties and maintained chemical stability. Moreover, HfO_2_ maintained the stability of the passive region and preserved its structural integrity after exposure and a reduction in anodic and cathodic current densities by up to three orders of magnitude. These findings establish ALD-deposited HfO_2_ films on SS316L as an effective corrosion barrier layer.

## Figures and Tables

**Table 1:** Corrosion parameters of SS316L and HfO_2_-coated SS316L obtained from potentiodynamic polarization measurements; $E_{corr}$—corrosion potential; j-current density at 50 mV; E_*pit*_—pitting potential. All potentials are reported vs. Ag|AgCl|Cl^−^ [mV].

Sample	E_*corr*_ [mV]	j [A·cm^−2^]	E_*pit*_ [mV]
SS316L	−107±8	$1.7\pm 0.1\times 10^{-6}$	-
HfO_2_	−247±47	$5.1\pm 2.2\times 10^{-10}$	678±196

**Table 2:** Electrochemical parameters of SS316L and HfO_2_ coating obtained from equivalent circuit fitting after 1 h in NaCl solution.

	R_*s*_	R_*pas*_	Q_*pas*_		R_*coat*_	Q_*coat*_	
			Y_0−*pas*_	n_*pas*_		Y_0−*coat*_	n_*coat*_
	Ω·cm^2^	MΩ·cm^2^	μS·s^*n*−1^·cm^−2^		GΩ·cm^2^	μS·s^*n*−1^·cm^−2^	
SS316L	50.1±0.2	0.23±0.01	28.3±0.5	–	–	–	–
HfO_2_	47.2±1.3	0.63±0.35	0.19±0.02	0.97±0.01	5.55±1.85	0.15±0.07	0.77±0.04

**Table 3:** The content of oxygen atoms in at.% of HfO_2_ coating obtained from O 1s line before and after PP test.

Samples		O_2_Hf	–OH	H_2_O
		**(at.%)**	**(at.%)**	**(at.%)**
HfO_2_	before PP	73.68	26.32	–
	after PP	44.42	44.03	11.55

## Data Availability

The original contributions presented in this study are included in the article. Further inquiries can be directed to the corresponding author.

## Abbreviations

The following abbreviations are used in this manuscript:

ALD	Atomic layer deposition
SS316L	Austenitic stainless steel 316L
PP	Potentiodynamic polarization
EIS	Electrochemical impedance spectroscopy
XPS	X-ray photoelectron spectroscopy
SEM	Scanning electron microscopy
AFM	Atomic force microscopy
TDMAH	Tetrakis(diethylamido) hafnium
CE	Counter electrode
RE	Reference electrode
WE	Working electrode
E_*OCP*_	Open-circuit potential
EEC	Electrochemical equivalent circuit
CPE	Constant phase element
FWHM	Full width at half maximum
BE	Binding energy
